# Genetic constraints for thermal coadaptation in *Drosophila subobscura*

**DOI:** 10.1186/1471-2148-10-363

**Published:** 2010-11-25

**Authors:** Olga Dolgova, Carla Rego, Gemma Calabria, Joan Balanyà, Marta Pascual, Enrico L Rezende, Mauro Santos

**Affiliations:** 1 Departament de Genètica i de Microbiologia, Grup de Biologia Evolutiva (GBE), Universitat Autònoma de Barcelona, 08193 Bellaterra (Barcelona), Spain; 2 Departamento de Ciências Agrárias, Azorean Biodiversity Group-CITAA, Universidade dos Açores, Terra-Chã, 9701-851 Angra do Heroísmo, Portugal; 3 Departament de Genètica, Grup de Biologia Evolutiva (GBE), Facultat de Biologia, Universitat de Barcelona, Diagonal 645, 08071 Barcelona, Spain; 4 Institut de Recerca de la Biodiversitat (IRBio), Universitat de Barcelona, 08071 Barcelona, Spain

## Abstract

**Background:**

Behaviour has been traditionally viewed as a driver of subsequent evolution because behavioural adjustments expose organisms to novel environments, which may result in a correlated evolution on other traits. In *Drosophila subobscura*, thermal preference and heat tolerance are linked to chromosomal inversion polymorphisms that show parallel latitudinal clines worldwide, such that "cold-climate" ("warm-climate") chromosome arrangements collectively favour a coherent response to colder (warmer) settings as flies carrying them prefer colder (warmer) conditions and have lower (higher) knock out temperatures. Yet, it is not clear whether a genetic correlation between thermal preference and heat tolerance can partially underlie such response.

**Results:**

We have analyzed the genetic basis of thermal preference and heat tolerance using isochromosomal lines in *D. subobscura*. Chromosome arrangements on the O chromosome were known to have a biometrical effect on thermal preference in a laboratory temperature gradient, and also harbour several genes involved in the heat shock response; in particular, the genes *Hsp68 *and *Hsp70*. Our results corroborate that arrangements on chromosome O affect adult thermal preference in a laboratory temperature gradient, with cold-climate O_st _carriers displaying a lower thermal preference than their warm-climate O_3+4 _and O_3+4+8 _counterparts. However, these chromosome arrangements did not have any effect on adult heat tolerance and, hence, we putatively discard a genetic covariance between both traits arising from linkage disequilibrium between genes affecting thermal preference and candidate genes for heat shock resistance. Nonetheless, a possible association of juvenile thermal preference and heat resistance warrants further analysis.

**Conclusions:**

Thermal preference and heat tolerance in the isochromosomal lines of *D. subobscura *appear to be genetically independent, which might potentially prevent a coherent response of behaviour and physiology (i.e., coadaptation) to thermal selection. If this pattern is general to all chromosomes, then any correlation between thermal preference and heat resistance across latitudinal gradients would likely reflect a pattern of correlated selection rather than genetic correlation.

## Background

Ectotherms exhibit a suite of behavioural and physiological strategies to cope with spatiotemporal variation in ambient temperature [1]. For instance, behavioural adjustments (e.g. modifying daily activity patterns and selecting favourable microclimates; [2]) can buffer the impact of sub-optimal temperatures, and are the main means of thermoregulation in small insects [3-5]. Although such adjustments can enable ectotherms to maintain relatively constant body temperatures (*T*_b_) at different seasons and/or latitudes [2,6], the observation of cyclical seasonal changes in genetic markers putatively related to thermal adaptation [7,8] and the clinal variation in thermal stress tolerance in some *Drosophila *species [9-11] suggest that behavioural thermoregulation may be insufficient to fully compensate shifts in environmental temperature [12].

If behavioural thermoregulation is not fully compensatory and climate variation influences the actual *T*_b _and physiological performance of organisms distributed over broad latitudinal ranges (i.e., performance falls below its optimum during cooling and warming), then temperature is more than just a key environmental factor that affects development, growth, and survival of individuals [13,14]: it is likely the main selective agent that drives - directly or indirectly - the evolution of clinal patterns in genetic, phenotypic, and life history traits. Furthermore, the divergence of thermal optima in the different subpopulations according to the *T*_b _experienced by the organism is expected to bolster a covariance between behavioural shifts (thermal preference) and performance [6,15]. This is related to the idea of "coadaptation" [16], where natural selection is supposed to favour the harmonious adjustment among the suite of (co-)evolving traits [7,17]. Parallel clines on different continents or along independent temperature gradients can thus offer an invaluable opportunity to study thermal coadaptation since the role of temperature in driving those clines is quite compelling.

Some widespread latitudinal clines in *Drosophila *also provide an additional advantage for studies of thermal coadaptation: there is a relatively well-known historical record following the invasion of a new geographical region (e.g. [18,19]). Perhaps the best example is that of *Drosophila subobscura*, a native Palaearctic species that invaded the Americas about 30 years ago, and spread rapidly on both South and North America. Clinal patterns for phenotypic traits and genetic polymorphisms emerged very rapidly during these two independent colonization events [20-22]. For instance, North American populations soon evolved decreased desiccation resistance with increasing latitude as expected, which matches the pattern found in Old World populations and suggests that strong selection for thermal-related traits along latitudinal gradients is taking place. On the other hand, in South America this trait shows the opposite pattern: higher desiccation tolerance is observed in colder areas [23]. Contrasting outcomes were also observed for other clinally varying traits - wing cell size and cell number [24], and wing shape [20,25] - where the role of temperature remains elusive, which apparently suggests that selective pressures vary in the different clines. An alternative explanation, however, is that evolution can sometimes be constrained by antagonistic genetic correlations (i.e., genetic correlations among traits that are not in accord with the direction of selection [26,27]) arising from linkage disequilibrium between alleles at different loci, and patterns of linkage disequilibrium can vary among populations or seasons [28,29]. In this context, we now know that contrasting wing shape clines in *D. subobscura *came out as a correlated response of the world-wide parallel inversion clines [21] because inversion-shape relationships in native and colonizing populations are opposite (presumably due to the different associations between inversions and particular alleles which influence the trait), probably as a result of the bottleneck effect that occurred during the colonization of America [30]. Besides, different patterns of linkage disequilibrium could result from variability in migration rates between genetically differentiated populations in the various latitudinal clines [31]. In summary, conflicting outcomes between old and rapidly evolving new clines should probably not be viewed as a nuisance, but as reminder that an appropriate knowledge of the underlying genetic architecture is required to further understand why (or why not) these inconsistencies arise. More specifically, if behaviour "drives" the subsequent parallel evolution in morphology and physiology as predicted ([6]; but see [32]), it is essential to analyze the genetic basis of thermal preference and temperature-related traits to see whether or not thermal coadaptation can happen along a cline.

We have recently undertaken a within-population large-scale study to analyze the association between chromosomal inversion polymorphisms that show parallel latitudinal clines in native and colonizing populations of *D. subobscura*, with the thermal preferences (*T*_p_: the preferred body temperature in a laboratory thermal gradient, which we expect to correlate with the thermal optimum for performance; [33]) and knock out temperatures (*T*_ko_: the temperature required to knock out a fly in a water-bath) of their carriers [34]. The main results can be summarized as follows: (*i*) flies carrying "cold-adapted" or "cold-climate" chromosome arrangements (i.e., those chromosome arrangements in all five major acrocentric chromosomes that show a negative correlation coefficient with maximum temperatures along the cline, or a positive correlation coefficient with latitude in Palaearctic populations; [35,36]) prefer a lower *T*_p _and had a lower *T*_ko_, in accordance with the natural patterns; (*ii*) different chromosomes were responsible for the bulk of the genetic variation in *T*_p _(chromosomes A and O) and *T*_ko _(chromosome E); and (*iii*) *T*_p _and *T*_ko _were phenotypically uncorrelated, which agrees with the observation that different independently segregating chromosomes were mainly responsible for the corresponding associations. Taken at a face value, behavioural thermoregulation and performance were indeed "coadapted" in the sense that cold-climate (warm-climate) chromosome arrangements collectively favour a coherent response to colder (warmer) environments, but this was not due to a genetic covariance of behaviour and physiology. There were, however, two potential limitations in the study. First, each individual fly was scored for only one chromosome of its diploid set and, hence, dominance effects (if any) where hidden in the analysis. Second, both intra- and interchromosomal contributions were mixed because the assayed flies had the genetic background from the sampled wild population. Although it might be argued that this protocol is somehow closer to what happens in nature, these uncontrolled factors might have precluded a better characterization of the underlying genetic effects. Accordingly, although the amount of genetic variation on *T*_p _and *T*_ko _explained by the combined effect of all chromosomes carrying at least one cold-climate gene arrangement was statistically significant, it only accounted for 1% of the total phenotypic variation [34].

**Figure 1:**
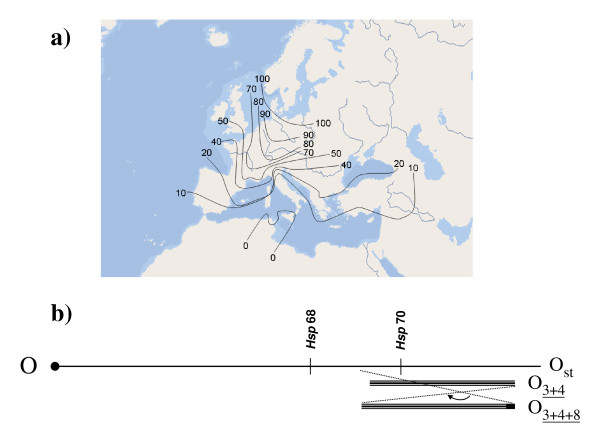
**Latitudinal cline of O_st _gene arrangement and schematic of chromosome O in *Drosophila subobscura***. (a) Lines in the Palaearctic region connect places at which O_st _depicts similar frequencies, and show a clear northwest-southwest cline (O_3+4 _shows an opposite cline). (b) Approximate location of genes *Hsp*68 and *Hsp*70 on chromosome O. The three gene arrangements used in the experiment are labelled on the right side of the schematic representation, with the centromere placed on the left (solid circle) and the telomere on the right. O_3+4 _consists of two overlapping inversions, and O_3+4+8 _of three.

Here we examine if *T*_p _and *T*_ko _are genetically correlated and might evolve in a coherent fashion in response to selection; i.e., whether behaviour and physiology are coadapted at the genetic level. We take advantage of the fact that the polymorphic inversions on chromosome O appear to be associated with behavioural thermoregulation in *D. subobscura *[34], and that this is the only chromosome that can be used to measure the expression of associated traits in replicated inbred and outbred genotypes. Namely, chromosome O is the only one for which a balancer stock (*Va*/*Ba*: *Varicose*/*Bare*; [37]) is available (a balancer is a specially constructed chromosome that carries a dominant morphological marker that is homozygous lethal and multiple inversions to suppress recombination). This is the longest chromosome in *D. subobscura *(190 cM which correspond to approximately 31 Mb [38]), and is homologous to arm 3R in *D. melanogaster *[39,40]. Some chromosome arrangements (O_st _and O_3+4_) show conspicuous northwest-southwest latitudinal clines in Palaearctic populations (Figure 1a). Chromosome O harbours several genes involved in the heat shock response [41]; in particular, gene *Hsp*68 (located in section O(89A) [42,43] and relatively close to the proximal breakpoint of inversion O_8 _[44]), and gene *Hsp*70 (located in section O(94A) [42,43] and included inside the warm-climate chromosomal arrangement O_3+4_, and close to the distal breakpoint of inversion O_8 _[44]) (Figure 1b). Hsp70 appears to be the primary protein involved in thermotolerance in *D. melanogaster *[45] - though apparently not in other *Drosophila *species [46] -, and *Hsp70 *allele frequencies show latitudinal clines and change in response to thermal evolution in the laboratory [47]. In addition, correlated responses to selection for knock down resistance at 39°C have also been found for *Hsp68 *in *D. melanogaster *[48].

Previous work also showed that *D. subobscura *flies carrying O chromosomes derived from replicated thermal lines [49,50] that had evolved in the laboratory at warm temperatures (22°C) had a higher total net fitness than its cold-adapted (13°C) counterparts; that is, a significant shift in thermal optima was observed [51]. All in all, it seems that there is indeed room for the coevolution of behaviour and physiological tolerance in *D. subobscura*. However, we show here that thermal preference and heat tolerance appear to be genetically independent. Therefore, any latitudinal correlation between both traits would likely reflect a pattern of correlated selection across populations rather than within-population genetic correlations.

## Experimental settings

In south-western European populations, the most frequent chromosome arrangements for chromosome O are O_st_, O_3+4_, O_3+4+7_, and O_3+4+8 _[52]. The first two arrangements show a clear contrasting clinal pattern in original Palaearctic populations, with O_st _increasing and O_3+4 _decreasing in frequency with increasing latitude [35,56] (Figure 1a). Arrangement O_3+4+8 _is also interesting because in historical times it was mainly restricted to the Mediterranean region, being the most abundant chromosomal arrangement in northern Africa [53]. However, in the last decades its distribution has changed dramatically and recent surveys revealed frequencies as high as 22.6% in Groningen, Netherlands, where it was previously absent [22,54]. Six independent isochromosomal lines for each of these three arrangements (i.e., ${\text{O}}_{j}^{\text{1}},...,{\text{O}}_{j}^{\text{6}}$; *j *= st, 3 + 4, 3 + 4 + 8) were used in the present experiments. Extensive genetic differentiation of up to 4 Mb (i.e., about 15% of the euchromatic portion) has been detected among these arrangements [55]. In other words, there are compelling reasons to think that the chromosome arrangements used in this work are genetically differentiated for *Hsp*70, and probably also for *Hsp68 *since inversion effects can extend as far as 1000 kilobases outside from breakpoints [56,57].

Following Santos et al. [58] the experimental flies were obtained from 54 crosses, which will be referred to as inbred (isogenic:${\text{O}}_{j}^{\text{1}}\times {\text{O}}_{j}^{\text{1}},{\text{O}}_{j}^{\text{2}}\times {\text{O}}_{j}^{\text{2}},...,{\text{O}}_{j}^{\text{6}}\times {\text{O}}_{j}^{\text{6}}$ with 18 crosses in total), or as outbred including both structural homokaryotypes (${\text{O}}_{j}^{\text{1}}\times {\text{O}}_{j}^{\text{2}},{\text{O}}_{j}^{\text{2}}\times {\text{O}}_{j}^{\text{3}},...,{\text{O}}_{j}^{\text{6}}\times {\text{O}}_{j}^{\text{1}}$ with 18 cyclically permuted reciprocal crosses in total) and heterokaryotypes (${\text{O}}_{j}^{\text{1}}\times {\text{O}}_{k}^{\text{1}},{\text{O}}_{j}^{\text{2}}\times {\text{O}}_{k}^{\text{2}},...,{\text{O}}_{j}^{\text{6}}\times {\text{O}}_{k}^{\text{6}}$; *j *≠ *k*; with 18 reciprocal crosses in total). Two developmental temperatures were used in the experiment to study potentially important effects of phenotypic plasticity: 18°C and 22°C. The reason for this was the huge difference (about 7°C-8°C) between our previous estimate of *T*_p _(pooled average 16.6°C; [34]) in *D. subobscura *flies raised at 18°C, and that obtained by Huey and Pascual (23.7°C; [12]) where flies were raised at 22°C. Even though the flies assayed came from different sources - south-western Europe in Rego et al. [34], and North America in Huey and Pascual [12] -, which could account for the observed difference because thermal responses can vary between populations [59], it remains to be seen whether developmental plasticity can affect estimates of thermal preference and heat tolerance.

## Results

### Association between thermal preference and knock out temperature

The phenotypic correlation between *T*_p _and *T*_ko _was assessed from their partial correlation coefficient, holding constant the variables developmental temperature, sex, plate hour, and water bath (see Methods). In no case were the partial correlations statistically significant: inbred crosses $r_{T_{\text{p}}\cdot T_{\text{ko}}}=0.065$, *t *= 1.21, df = 347, *P *= 0.226; outbred crosses $r_{T_{\text{p}}\cdot T_{\text{ko}}}=-0.030$, *t *= 0.79, df = 701, *P *= 0.429. Furthermore, as expected from the low values of the phenotypic correlation, the genetic (karyotypic) correlation for the outbred flies was also close to zero (*r_k _*= -0.068, *P *= 0.914). The conclusion is that both traits are nearly orthogonal to each other (pooled $r_{T_{\text{p}}\cdot T_{\text{ko}}}=1.2\times 10^{-4}$, *t *= 0.004, df = 1054, *P *= 0.997) and, hence, they will be analyzed separately in what follows.

### Consanguinity and developmental effects

#### a) Thermal preference

**Figure 2:**
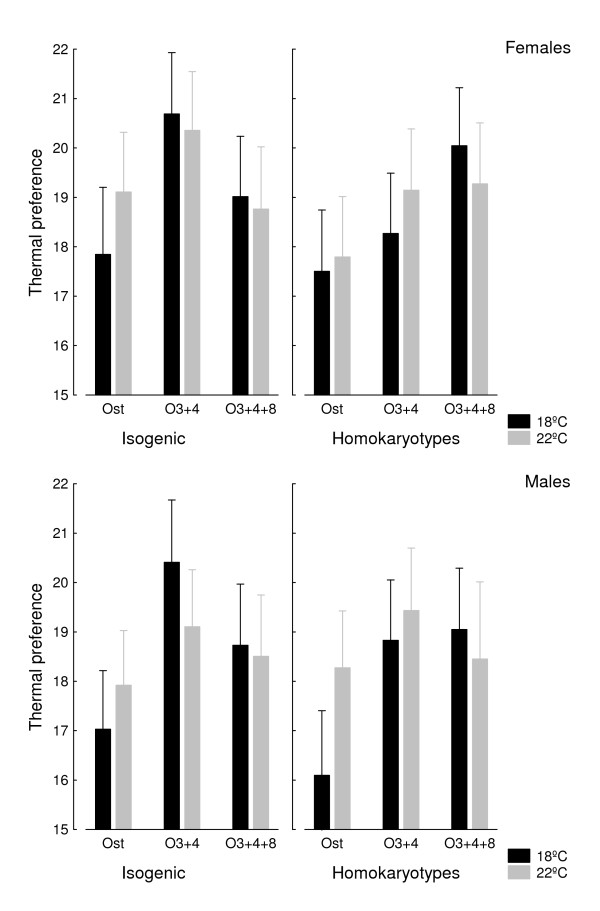
**Inbreeding and temperature effects on thermal preference**. Homokaryotipic averages for *T*_p _(in °C with 95% confidence intervals) in inbred (left panels) and outbred (right panels) crosses according to sex and developmental temperature.

Inbreeding and developmental temperature effects on *T*_p _were simultaneously analyzed by contrasting isogenic *vs*. outbred homokaryotypic flies reared at both experimental temperatures (Figure 2). The factorial analysis of covariance (ANCOVA) only detected statistically significant differences for karyotypes, karyotype × inbreeding interaction, and karyotype × developmental temperature interaction effects (Table 1). Average (± SD) *T*_p _was not different between rearing temperatures (flies reared at 18°C: 18.7°C ± 4.1°C; flies reared at 22°C: 18.8°C ± 3.1°C) or sexes (females: 19.0°C ± 3.6°C; males: 18.5°C ± 3.6°C), although in this last case the effect was marginally non-significant (*P *= 0.053). Permutation tests (see Methods) corroborated that the three assayed karyotypes differ in *T*_p _(P = 0.001).

**Table 1:** Inbreeding and temperature effects on thermal preference.

Source of variation	**d.f**.	Mean Square	*F*	*P*
Covariate (plate hour)	1	25.502	2.07	0.151
Karyotype (*κ*)	2	231.515	18.29	< 0.001
Cross ⊂ *κ*	15	12.676	1.03	0.425
Inbreeding (*ι*)	1	30.514	2.47	0.116
Temperature (*τ*)	1	4.119	0.33	0.564
Sex (*ς*)	1	46.227	3.74	0.053
*κ *× *ι*	2	40.337	3.27	0.039
*κ *× *τ*	2	40.031	3.24	0.040
*κ *× *ς*	2	6.195	0.50	0.606
*ι *× *τ*	1	11.063	0.90	0.344
*ι *× *ς*	1	6.257	0.51	0.477
*τ *× *ς*	1	0.408	0.03	0.856
*κ *× *ι *×* τ*	2	17.477	1.42	0.243
*κ *× *ι *× *ς*	2	11.532	0.93	0.393
*κ *× *τ *×* ς*	2	7.600	0.62	0.541
*ι *× τ × *ς*	1	12.123	0.98	0.322
*κ *× *ι *× τ ×*ς*	2	4.245	0.34	0.709
Error	717	12.346		

Flies raised from inbred (isogenic) and outbred crosses of *Drosophila subobscura *reared at 18°C and 22°C. Karyotypes being compared are O_st_/O_st_, O_3+4_/O_3+4_, and O_3+4+8_/O_3+4+8_. (⊂ means "nested in".)

Scheffé post hoc tests using the mean square of the nested "cross" effect as the error term showed that the thermal preference of O_st_/O_st _flies was significantly lower when compared to those of O_3+4_/O_3+4 _and O_3+4+8_/O_3+4+8 _homokaryotypes, which did not differ between them. The difference is consistent for both isogenic and outbred flies (Figure 2). From the present data we can conclude that the preferred temperature ranges or "set point" (*T*_set_) ranges (central 50% of preferred body temperatures; [60]) are bounded by 15.1°C - 20.5°C for O_st_/O_st _karyotypes, and 16.6°C - 22.2°C for the other two karyotypes.

The karyotype × inbreeding interaction arises from the somewhat different behaviour between O_st_/O_st _and O_3+4_/O_3+4 _karyotypes on one side, and O_3+4+8_/O_3+4+8 _on the other: for the first two karyotypes *T*_p _was slightly higher in inbred crosses when compared to their outbred counterparts, whereas the opposite was true for the O_3+4+8_/O_3+4+8_ karyotype. Average *T*_p _was, however, almost identical for inbred (18.9°C ± 3.6°C) and outbred (18.5°C ± 3.6°C) flies. On the other hand, O_st_/O_st _flies raised at 22°C had a higher *T*_p _than those raised at 18°C, but no clear trend was observed for O_3+4_/O_3+4 _and O_3+4+8_/O_3+4+8 _karyotypes.

#### b) Knock out temperature

**Figure 3:**
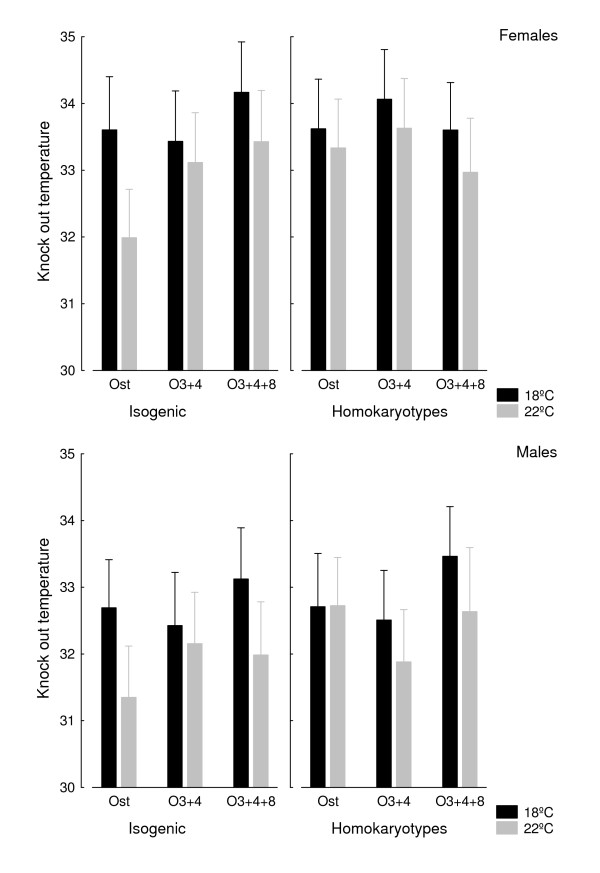
**Inbreeding and temperature effects on knock out temperature**. Homokaryotipic averages for *T*_ko _(in °C with 95% confidence intervals) in inbred (left panels) and outbred (right panels) crosses according to sex and developmental temperature.

Knock out temperatures are plotted in Figure 3. The ANCOVA (Table 2) detected statistically significant differences for the effects of rearing temperature and sex. Flies reared at 18°C had a higher *T*_ko _than flies reared at 22°C (mean ± SD: 33.3°C ± 2.1°C *vs*. 32.6°C ± 2.3°C), and females had a higher *T*_ko _than males (33.4°C ± 1.9°C *vs*. 32.5°C ± 2.4°C). Even though *T*_ko _was slightly lower for the isogenic lines when compared to their outbred counterparts (32.8°C ± 2.2°C *vs*. 33.1°C ± 2.2°C), inbreeding effects were clearly non-significant (P = 0.136).

**Table 2:** Inbreeding and temperature effects on knockout temperature.

Source of variation	**d.f**.	Mean Square	*F*	*P*
Covariate (water bath)	1	103.117	24.04	< 0.001
Karyotype (κ)	2	3.878	0.36	0.704
Cross ⊂ κ	15	11.027	2.57	0.001
Inbreeding (*ι*)	1	9.538	2.22	0.136
Temperature (τ)	1	77.034	17.96	< 0.001
Sex (*ς*)	1	154.979	36.13	< 0.001
*κ *× *ι*	2	4.176	0.97	0.378
*κ *× *τ*	2	1.999	0.47	0.628
*κ *× *ς*	2	8.106	1.89	0.152
*ι *× τ	1	1.047	0.24	0.621
*ι *× *ς*	1	0.435	0.10	0.750
*τ *× *ς*	1	0.022	0.01	0.943
*κ *× *ι *×*τ*	2	7.798	1.82	0.163
*κ *× *ι *× *ς*	2	8.926	2.08	0.126
*κ *× *τ *×*ς*	2	1.693	0.39	0.674
*ι *×τ × *ς*	1	0.241	0.06	0.813
*κ *× *ι *× τ × *ς*	2	0.159	0.04	0.964
Error	668	4.289		

Flies raised from inbred (isogenic) and outbred crosses of *Drosophila subobscura *reared at 18°C and 22°C. Karyotypes being compared are O_st_/O_st_, O_3+4_/O_3+4_, and O_3+4+8_/O_3+4+8_. (⊂ means "nested in".)

### Gene arrangement effects in the outbred lines

#### a) Thermal preference

The genetic and environmental (developmental temperature) contributions of chromosome O to *T*_p _(and *T*_ko_; below) was assessed from the outbred crosses including all possible karyotypes. Outbred crosses are obviously more relevant to the real situation because inbred genotypes are homozygous for deleterious alleles, and also for alleles that might display heterozygote advantage in the original outbred population. The only statistically significant effects detected by the ANCOVA model (Table 3) were those arising from genetic differences among karyotypes (permutation tests corroborated that the three assayed karyotypes differ in *T*_p_; *P *= 0.0018) and sexes, with females having a higher *T*_p _(mean ± SD: 18.7°C ± 3.6°C) than males (18.0°C ± 3.6°C). As above, average *T*_p _was slightly lower for flies reared at 18°C (18.1°C ± 4.0°C) than at 22°C (18.6°C ± 3.2°C), but the difference was marginally non-significant (*P *= 0.069).

**Table 3:** Karyotype and temperature effects on thermal preference.

Source of variation	**d.f**.	Mean Square	*F*	*P*
Covariate (plate hour)	1	147.947	11.84	< 0.001
Karyotype (*κ*)	5	60.774	4.97	0.002
${\text{O}}_{\text{st}}/{\text{O}}_{3+4}^{*}$	1	0.853	0.07	0.793
${\text{O}}_{3+4}^{*}/{\text{O}}_{3+4}^{*}$	2	42.884	3.51	0.043
${\text{O}}_{\text{st}}/{\text{O}}_{\text{st}},{\text{ O}}_{\text{st}}/{\text{O}}_{3+4}^{*}$, ${\text{O}}_{3+4}^{*}/{\text{O}}_{3+4}^{*}$	2	106.330	8.70	0.001
additive effect	1	205.854	16.85	< 0.001
dominance effect	1	3.532	0.29	0.595
Cross ⊂ *κ*	30	12.220	0.98	0.502
Temperature (*τ*)	1	41.328	3.31	0.069
Sex (*ς*)	1	91.221	7.30	0.007
*κ *× τ	5	19.791	1.58	0.162
*κ *× *ς*	5	10.805	0.86	0.505
τ × *ς*	1	4.948	0.40	0.529
*κ *× τ × *ς*	5	8.863	0.71	0.617
Error	691	12.498		

Flies raised from outbred crosses of *Drosophila subobscura *reared at 18°C and 22°C. Karyotypes being compared are O_st_/O_st_, O_3+4_/O_3+4_, O_3+4+8_/O_3+4+8_, O_st_/O_3+4_, O_st_/O_3+4+8 _and O_3+4_/O_3+4+8_. ${\text{O}}_{3+4}^{*}$ stands for O_3+4 _+ O_3+4+8_. (⊂ means "nested in".)

**Figure 4:**
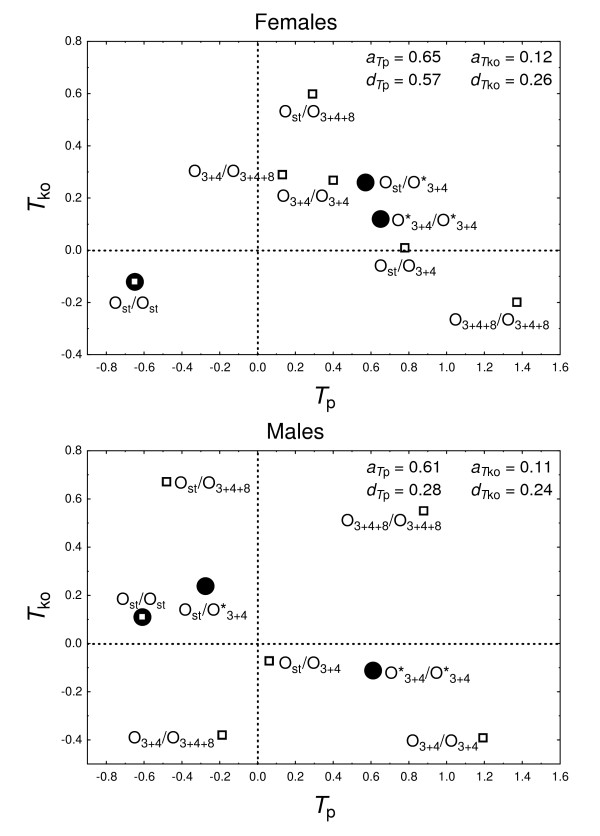
**Karyotypic values in the additive-dominance scale**. Deviation values for thermal preference (*T*_p_) and knockout temperature (*T*_ko_) were measured after pooling arrangements O_3+4 _and O_3+4+8 _into a single class (${\text{O}}_{3+4}^{*}$), and the coordinate point (0, 0) was taken as the midparent (i.e., the average of *T*_p _and *T*_ko _for the two karyotypes O_st_/O_st _and ${\text{O}}_{3+4}^{*}/{\text{O}}_{3+4}^{*}$). Females (upper panel) and males (lower panel) are plotted separately because the interaction karyotype × sex was statistically significant for *T*_ko _(Table 4). In the original scale the (0, 0) point corresponds to an average *T*_p _of 18.31°C for females and 17.91°C for males, and an average *T*_ko _of 33.58°C for females and 32.61°C for males. Open squares give the values for all six karyotypes to appreciate their dispersion from the midparent, as well as their dispersion from the pooled ${\text{O}}_{\text{st}}/{\text{O}}_{3+4}^{*}$ and ${\text{O}}_{3+4}^{*}/{\text{O}}_{3+4}^{*}$ karyotypes (black circles). Statistical significance for additive ($a_{T_{\text{p}}}\text{,}a_{T_{\text{ko}}}$) and dominance ($d_{T_{\text{p}}}\text{,}d_{T_{\text{ko}}}$) effects are given in Tables 3 and 4. Note also that the phenotypic ($r_{T_{\text{p}}\cdot T_{\text{ko}}}=-0.030$) and genetic (*r_k _*= -0.068, *r_p _*= -0.130; see Methods) correlations were non-significantly different from zero (see text for details).

The linear contrast between the two ${\text{O}}_{\text{st}}/{\text{O}}_{3+4}^{*}$ heterokaryotypes (${\text{O}}_{3+4}^{*}$ pools into a single class the arrangements that share O_3+4_; see Methods) reveals that O_st_/O_3+4 _and O_st_/O_3+4+8 _flies displayed a similar average *T*_p _(18.5°C ± 3.8°C *vs*. 18.0°C ± 3.7°C, respectively). However, some differences were detected among the three ${\text{O}}_{3+4}^{*}/{\text{O}}_{3+4}^{*}$ karyotypes, which can be attributed to some under-dominance because average *T*_p _for O_3+4_/O_3+4+8 _flies (18.1°C ± 3.4°C) was lower than that for the corresponding homokaryotypes (O_3+4_/O_3+4_: 18.9°C ± 3.5°C; O_3+4+8_/O_3+4+8_: 19.3°C ± 3.6°C). In any case, the main difference was between O_st _and ${\text{O}}_{3+4}^{*}$ carriers, with mainly additive genetic effects (Figure 4). As already indicated, O_st_/O_st _flies clearly preferred lower temperatures than O_3+4_/O_3+4 _or O_3+4+8_/O_3+4+8 _flies.

#### b) Knock out temperature

The ANCOVA for *T*_ko _(Table 4) did not detect any difference among karyotypes, in accordance with the previous findings for the inbred crosses. Similarly, the main differences arose between developmental temperature (flies reared at 18°C: 33.6°C ± 1.9°C; flies reared at 22°C: 32.8°C ± 2.3°C) and sex (females: 33.7°C ± 1.8°C; males: 32.7°C ± 2.4°C).

**Table 4:** Karyotype and temperature effects on knockout temperature.

Source of variation	**d.f**.	Mean Square	*F*	*P*
Covariate (water bath)	1	101.377	25.87	< 0.001
Karyotype (*κ*)	5	4.295	0.57	0.724
${\text{O}}_{\text{st}}/{\text{O}}_{3+4}^{*}$	1	11.598	1.52	0.228
${\text{O}}_{3+4}^{*}/{\text{O}}_{3+4}^{*}$	2	0.016	0.002	0.998
${\text{O}}_{\text{st}}/{\text{O}}_{\text{st}},{\text{ O}}_{\text{st}}/{\text{O}}_{3+4}^{*}$, ${\text{O}}_{3+4}^{*}/{\text{O}}_{3+4}^{*}$	2	4.872	0.64	0.536
additive effect	1	0.015	0.001	0.965
dominance effect	1	8.632	1.13	0.296
Cross ⊂ *κ*	30	7.641	1.95	0.002
Temperature (*τ*)	1	107.075	27.33	< 0.001
Sex (*ς*)	1	180.874	46.16	< 0.001
*κ *× *τ*	5	7.576	1.93	0.087
*κ *× *ς*	5	8.777	2.24	0.049
*τ *× *ς*	1	1.650	0.42	0.517
*κ *× τ × *ς*	5	2.329	0.59	0.704
Error	654	3.918		

Flies raised from outbred crosses of *Drosophila subobscura *reared at 18°C and 22°C. Karyotypes being compared are O_st_/O_st_, O_3+4_/O_3+4_, O_3+4+8_/O_3+4+8_, O_st_/O_3+4+8_, and O_3+4_/O_3+4+8_. ${\text{O}}_{3+4}^{*}$ stands for O_3+4 _+ O_3+4+8_. (⊂ means "nested in".)

The genetic correlation between *T*_p _and *T*_ko _after pooling O_3+4 _and O_3+4+8_was *r_p _*= -0.130 (P = 0.917). Again, the conclusion is that these two traits are uncorrelated. Figure 4 plots the genotypic values in the additive-dominance scales for *T*_p _and *T*_ko_, together with their statistical significance obtained from the appropriate contrasts (Table 3, 4).

#### c) Average effects on thermal preference

Our experiment only provides an estimation of the gene (chromosome O) action on *T*_p _and does not allow inferences to the base population. It is possible, however, to obtain estimates of the average effects, or "statistically additive effects", by taking into account the gene action and allelic (chromosome arrangement) frequencies in the natural populations [61]. Assuming that the chromosome arrangement effects are roughly the same along the cline (for a measure of climatic temperatures along the Palaearctic cline see Figure 1 in [62]), Table 5 gives the average effects (females and males pooled) estimated from the frequencies of the different arrangements in European populations spanning about 17° latitude [52,54]. The interpretation is that flies inheriting a O_st _chromosome will choose a temperature ranging from around 0.31°C - 0.45°C below the average temperature chosen by the population (conversely, flies carrying warm-climate chromosome arrangements will choose a temperature ranging from around 0.03°C - 0.52°C above the average).

**Table 5:** Average effect of chromosome O on thermal preferences (°C).

		Frequency		Average effect	
Population	Coordinates	**O**_**st**_	${\text{O}}_{3+4}^{*}$	**O**_**st**_	Rest
Málaga (Spain)	36°43'N - 4°25'W	0.080	0.407	-0.4506	0.0392
Punta Umbría (Spain)	37°10'N - 6°57'W	0.066	0.410	-0.4494	0.0318
Calviá (Spain)	39°33'N - 2°29'E	0.057	0.590	-0.4485	0.0271
Riba-roja (Spain)	39°33'N - 0°34'W	0.148	0.324	-0.4530	0.0787
Queralbs (Spain)	42°13'N - 2°10'E	0.290	0.493	-0.4395	0.1795
Lagrasse (France)	43°05'N - 2°37'E	0.330	0.590	-0.4312	0.2124
Montpellier (France)	43°36'N - 3°53'E	0.362	0.557	-0.4232	0.2401
Villars (France)	45°26'N - 0°44'E	0.389	0.581	-0.4155	0.2645
Leuk (Switzerland)	46°19'N - 7°39'E	0.595	0.365	-0.3267	0.4800
Vienna (Austria)	48°13'N - 16°22'E	0.625	0.270	-0.3095	0.5158
Tübingen (Germany)	48°32'N - 9°04'E	0.606	0.351	-0.3205	0.4930
Louvain-la-Neuve (Belgique)	50°43'N - 4°37'E	0.397	0.540	-0.4130	0.2719
Groningen (The Netherlands)	53°13'N - 6°35'E	0.502	0.405	-0.3733	0.3763

${\text{O}}_{3+4}^{*}$ pools gene arragements O_3+4 _and O_3+4+8 _used in the present work. Together with O_st_, their combined frequency is ≥ 0.90 in central European populations and drops to approximately 0.50 in south-western Europe, where arrangement O_3+4+7 _is also frequent. However, from our previous data [34] no difference in *T*_p _is detected between O_3+4+7 _and ${\text{O}}_{3+4}^{*}$, which justifies their pooling and allows estimating average effects assuming two gene arrangements: O_st _and the rest. Gene arrangement frequencies where taken from the "new collections" in Solé et al. [52] and Balanyà et al. [54].

Combined with our previous results with chromosome A (which is the sex chromosome and additive values can be estimated using males' *T*_p_; [34]), where gene arrangement A_st _exhibits a similar latitudinal pattern than O_st _and flies carrying A_st _also display a laboratory thermal preference towards colder temperature, the conclusion is that flies inheriting simultaneously A_st _and O_st _will choose temperatures ranging from approximately 0.5°C - 1.0°C below the average (these estimates assume perfect additivity).

## Discussion

The present results with isogenic lines and their crosses corroborate and extend our previous work with wild flies from south-western Europe [34]. They confirm that arrangements on chromosome O have a biometrical effect on thermal preference in a laboratory temperature gradient, with cold-climate O_st _carriers displaying a lower *T*_p _than their warm-climate O_3+4 _and O_3+4+8 _counterparts. In addition, *T*_p _and *T*_ko _were again found to be uncorrelated, and we can now discard a potential genetic covariance between both traits arising from linkage disequilibrium between genes affecting thermal preference and candidate genes for heat shock resistance (i.e., *Hsp*68 and *Hsp70*; [42,43]) located inside of, or close to, the chromosome regions covered by the inversions analyzed here (see Background). In other words, we conclude that variation on O chromosome arrangements does not have any effect on knock out temperature (but see below). Note, however, that this does not imply that genes on chromosome O have no effect on *T*_ko _(actually, statistically significant differences were detected among crosses within karyotypes; Table 4); it simply indicates that any allelic variation of putative genes influencing this trait is not in linkage disequilibrium with inversions on this chromosome.

The new findings were: (*i*) a lack of inbreeding depression for both *T*_p _and *T*_ko_; (*ii*) a lack of phenotypic plasticity for *T*_p _according to the temperature at which the flies were raised (18°C and 22°C); and (*iii*) a substantial effect of developmental temperature on *T*_ko_. The absence of inbreeding depression for *T*_p _agrees with the genetic analysis from outbred flies, where a dominance effect after pooling chromosome arrangements O_3+4 _and O_3+4+8 _into a single class (${\text{O}}_{3+4}^{*}$) was absent (Table 3; note that the differences detected among the three ${\text{O}}_{3+4}^{*}/{\text{O}}_{3+4}^{*}$ karyotypes, and attributed to some under-dominance, could not be appreciated in the inbreeding analysis because it only included inbred and outbred homokaryotypes). On the other hand, the lack of inbreeding depression for *T*_ko _is expected and does not mean anything here, simply because no "gene" effects linked to chromosomal arrangements on chromosome O were detected. At first sight this might be surprising because a well-characterized cellular defence mechanism once environmental temperature approaches the upper thermal limits is the heat shock response, and in *D. melanogaster *the major inducible heat shock protein Hsp70 appears to be the primary protein involved in thermotolerance [45,63]. Recent work, however, questions the pervasive role of Hsp70 in the mediation of the heat stress response and suggests that it may be life-stage specific, being important in larvae but not in adults [64]. Our results are apparently consistent with the lack of association between Hsp70 and adult heat resistance (but see further discussion below), although also raise a caveat to the conclusion that there is no covariance between *T*_p _and *T*_ko_. Thus, it could be the case that Hsp70 variation across karyotypes is associated with juvenile tolerance to heat stress, an important trait in *Drosophila *particularly in summer when larval feeding patches can become lethally hot [65]. This possibility warrants further analysis.

An important concern here is that Hsp70 production might not be inducible in the dynamic experimental protocol we used to estimate upper thermal tolerance, where temperature increased 0.1°C min^-1^. One apparently compelling reason for this is that the estimated maximum thermal limits that *D. melanogaster *can tolerate decrease from approximately 39.9°C with heating rate 0.5°C min^-1 ^to 38.7°C with heating rate 0.1°C min^-1 ^[66], a puzzling result because slower heating rates should allow individuals to acclimatize to new temperatures and also because slow heating rates pre-exposes individuals to non-lethal high temperatures ("hardening"), which increases heat shock resistance [10]. We have recently discussed why these conflicting outcomes arise, and suggest that the contribution of other stressors (e.g. higher desiccation in long thermal tolerance assays associated with slow warming rates) can potentially overshadow thermal acclimation effects in dynamic assays with varying heating rates [67]. In other words, we challenge the idea that induced thermotolerance does not occur in dynamic assays with slow heating rates. At this stage this is just speculative because Hsp70 production was not measured in our flies, but the problem is important because *Drosophila *adults are likely to experience slow heating rates in nature of 0.06 - 0.1°C min^-1 ^[66,68] and further empirical studies are required to explain the apparently inconsistent findings.

The pooled average *T*_p _here was (mean ± SD) 18.4°C ± 3.6°C (*T*_set_: 15.4°C - 21.2°C; these figures include only outbred lines) and about the same at both rearing temperatures. The difference with our previous estimate for wild-flies from Adraga (16.6°C, *T*_set_:12.4°C - 20.4°C; [34]) does not seem to be overreached, and could be partially explained by the fact that the present flies were genetically homogeneous for all chromosomes from the *ch*-*cu *marker strain but chromosome O (recall that the sex chromosome A also had a significant effect on *T*_p_; [34]). This strain has a long history of maintenance at 18°C in the laboratory. In any case, our estimates remain substantially lower than that from Huey and Pascual (23.7°C, *T*_set_: 21.2°C - 25.9°C; [12]), and the difference cannot be accounted by flies' rearing temperature. No reasonable explanation for the discrepancy can be offered at this moment, but the additional result that developmental temperature substantially affected *T*_ko _makes us confidently conclude that our estimates are indeed closer to the actual *T*_p _of the species. Flies reared at 22°C showed lower heat resistance than their counterparts reared at 18°C (32.8°C *vs*. 33.6°C; outbred lines), which could be a consequence of their smaller size due to the inverse relationship between body size and developmental temperature [69,70]. However, resistance to heat does not seem to be associated with body size [71] - we have also analyzed the association between *T*_ko _and wing size from our previous experiment where both traits were recorded [30,34] and found no relationship whatsoever (results not shown). Most likely, 22°C was a suboptimal and potentially stressful temperature for our flies, making them to be weaker and less resistant to the heat shock. Note, however, that this conclusion might not be extrapolated to wild flies that harbour higher levels of genetic variability than our chromosomal lines.

To interpret the interplay between thermal preference and heat stress resistance, an understanding of the environmental temperatures experienced by *D. subobscura *along climatic gradients is required. As far as we are aware, the only data available on *T*_b _for active flies along a latitudinal gradient (spanning 12°) come from recent work by Huey and Pascual [12] in western North America. They found that mean *T*_b _varies by as much as 21°C (from 8°C to 29°C), and that the temporal activity of flies during the day did not match predictions from optimal temperature regulation or desiccation avoidance. Temperatures of maximum activity in summer (Figure 2 in [12]) - when wild flies are smaller probably due to their higher developmental temperatures and/or crowding conditions [72]; and crowding is known to affect adult thermal stress resistance in *Drosophila *[73] - are dangerously close to the *T*_ko _obtained here for the outbred flies raised at 22°C. This suggests that active *D. subobscura *flies can experience extreme conditions in the wild, and one would expect flies' activity to be correlated with heat resistance under these conditions if behaviour and physiology were coadapted. Some evidence indicates that diurnal activity patterns in summer can vary according to inversion polymorphism, and chromosome arrangements on the O chromosome seem to behave as expected from our data: O_st _is more frequent towards the evening while chromosomes carrying gene arrangement O_3+4 _are most frequently sampled at midday [74]. This behavioural thermoregulation, however, would not confer less susceptibility to high temperatures because the genetic basis of both traits does not seem to allow for the building up of "coadaptation". It is well known from basic population genetics theory that genetic covariance between traits can arise when alleles at different loci are associated (linkage disequilibrium), and this critically depends on relatively low recombination rates [75]. The lack of association between *T*_p _and *T*_ko _in *D. subobscura *is fully consistent with their genetic basis as independently segregating chromosomes are involved [34]. Yet, a correlation between these traits can be expected at the interpopulational level due to patterns of correlated selection (rather than genetic correlations) across a latitudinal gradient because of the congruent latitudinal clinal variation for chromosome arrangements on the E (which influences *T*_ko _[34]), and on the A and O chromosomes (which influence *T*_p _[[34], this work]).

We now speculate that the mismatch between *T*_p _and *T*_ko _could apparently generate an interesting dynamics in the population frequencies of different chromosome arrangements on chromosome O. Suppose the daily activity of flies in the warmest months of the year follows the previously described pattern; that is, flies carrying gene arrangement O_3+4 _are more active at midday and, therefore, have a higher risk of a heat shock than O_st _and are selected against. On the other hand, assuming *T*_p _corresponds closely with temperatures that maximize fitness O_3+4 _flies likely enjoy a fitness advantage in summer. The net effect would be a compromise between "behaviour unresponsiveness" and general performance, which means that chromosome arrangements on chromosome O may or may not cycle seasonally according to average environmental temperature (i.e., O_3+4 _could be expected to increase in frequency in summer and decrease in winter if general performance is what matters). Interestingly, both patterns have been detected: consistent seasonal cycling at a north-western population in Spain [8] and apparently no seasonal variation at a north-eastern population also in Spain [76]. The point here is that parallel seasonal changes should also be detected for chromosome A since it also affects *T*_p _[34]. In accordance with this prediction, no seasonal cycling was detected for chromosome A in the north-eastern population, but unfortunately no information is available for the other population because chromosome O was the only chromosome scored. It would be very interesting to see what happens for chromosome A in the cycling population.

## Conclusions

For ectotherms facing spatiotemporal variation in environmental temperature theory predicts that a coevolution between thermal preference and physiological performance can occur [1]. In the widespread species *D. subobscura *behavioural thermoregulation and heat tolerance are "coadapted" in the sense that flies carrying cold-climate (warm-climate) chromosome arrangements tend to choose colder (warmer) temperatures and have lower (higher) heat stress tolerance [34]. We have analyzed the genetic basis of these thermal traits using isochromosomal lines for the O chromosome. This chromosome was known to affect thermal preference [34], and also harbours several genes involved in the heat shock response (*Hsp68 *and *Hsp70*) [42,43]. These genes are located inside of, or close to, the chromosome regions covered by inversions that show conspicuous northwest-southwest latitudinal clines in Palaearctic populations, as well as seasonal fluctuations that are in agreement with the latitudinal patterns [22]. Our results corroborate that arrangements on chromosome O affect adult thermal preference: flies inheriting the cold-climate O_st _chromosome are predicted to choose a temperature around 0.31°C - 0.45°C below the average temperature chosen by the population and, conversely, flies inheriting the warm-climate O_3+4 _and O_3+4+8 _chromosomes are expected to choose a temperature ranging from around 0.03°C - 0.52°C above the average. However, these chromosome arrangements did not have any differential effect on adult heat tolerance. We conclude that thermal preference and heat tolerance in *D. subobscura *appear to be genetically independent and, therefore, any latitudinal correlation between both traits would likely reflect a pattern of correlated selection across populations rather than within-population genetic correlations.

## Methods

### Origin of flies and experimental procedures

*D. subobscura *wild flies were collected near Barcelona (41°43'N, 2°13'E) in October 2007. More than 200 isofemale lines were derived and used to obtain isochromosomal lines for the O chromosome in an otherwise homogeneous genetic background following standard protocols [77]. Briefly, one offspring male from each isofemale line was crossed to three or four virgin females from the *ch*-*cu *marker strain, which is homozygous for the morphological recessive markers on the O chromosome *cherry *eyes (*ch*) and *curled *wings (*cu*) and the chromosomal arrangement O_3+4_. A single wild-type male from each cross was repeatedly backcrossed to three or four *ch*-*cu *females for at least five generations in order to homogenize the genetic background, and the chromosomal arrangement carried by the wild chromosome was identified after the second backcross. To derive the isochromosomal lines, wild-type males from each line were crossed with the *Va*/*Ba *balancer stock [78], which has the same genetic background as the *ch*-*cu *strain. Once obtained, the isochromosomal lines were genotyped for 13 microsatellite loci located on the O chromosome to check that no recombination events occurred during the different crosses. The 18 independent isochromosomal lines used in this study (see Experimental settings) were found to be homozygous for all the loci. The lines were kept at 18°C (12:12 light/dark cycle) in 130-mL bottles with low adult density (around 20 pairs/bottle) to standardize the rearing conditions before egg collections.

To obtain the experimental flies, all 54 crosses (inbred and outbred) were performed at 18°C by mating 4 days-old virgin males and females from the corresponding isochromosomal lines. After six days the males were discarded and the females (an equal number from each reciprocal cross in the outbred combinations) were transferred to egg-laying chambers containing fresh food and charcoal colouring. Eggs were placed in vials (45 eggs/vial containing 6 mL of food) at two rearing temperatures: 18°C and 22°C. Non-anaesthetized emerging flies were stored in bottles at low adult density and used to evaluate laboratory thermal preference (*T*_p_) and knock out temperature (*T*_ko_) for each cross (see below). All fly handling was done at room temperature using CO_2 _anaesthesia only to sort virgin flies and to place females in the egg-laying chambers.

### Thermal preference behaviour in a laboratory gradient and heat resistance

Laboratory *T*_p _was measured as previously described [34]. Briefly, adult flies (about 7 days old) were individually placed in separate lanes on an aluminium base plate where a thermal gradient with temperatures ranging from 11°C to 29°C was generated. Adults were given approximately 1 h to adjust, and afterwards their positions were recorded four times every 10 min. We used the median of the four measurements to estimate *T*_p _of each fly. Measurements were performed in a room with constant temperature (22°C - 23°C), and the flies were assayed under white light illumination. This protocol renders a repeatable assessment of flies' thermal preferences [34]. After the thermal preference assay, each fly was gently removed from the lane and individually placed in a vial with fresh food for the subsequent assay of heat stress tolerance.

One day after measurements of thermal preference flies were assayed for heat resistance also as previously described [34]. Adults were individually placed in sealed empty vials and immersed in water-baths at *T*_min _= 24°C. Every 10 min individuals were scored for mobility (fly active or knocked out) and the temperature of the water was increased by Δ*T *= +1°C. The procedure was repeated until the water-baths reached *T*_max_, defined as the temperature when the last active fly was knocked out (*T*_max _= 38°C was the upper limit in the assays; median *T*_max _= 33°C). For each fly *T*_ko _was estimated as the temperature taken to knock it out (defined as the onset of muscle spasms; [79]).

### Statistical methods

The experimental setup was devised to assay one male and one female from each cross and temperature per day (five blocks) for both *T*_p _and *T*_ko_, amounting to 1,080 flies in total. Some mishaps (e.g. individuals flew away or just died during the assays) were, however, unavoidable and the final data set contains a few more than or a few less than 10 flies in several crosses (the harmonic means of flies per cross and temperature were: *T*_p _assay, 5.04 females and 4.80 males; *T*_ko _assay, 4.89 females and 4.37 males). Statistical analysis with and without block design qualitatively yielded the same results. Therefore, to simplify matters blocks were not considered in the linear models below.

#### a) Consanguinity and temperature effects

Inbreeding and temperature effects were simultaneously analyzed by contrasting isogenic *vs*. outbred homokaryotypic flies reared at both developmental temperatures. The linear model used was:

(1) Tp(ijklmn)=μ+κi+Cj(i)+τk+ιl+ςm+κτik+κιil+κςim+τιkl+τςkm+...+ιςlm+κτιikl+κτςikm+κιςilm+τιςklm+κτιςiklm+εijklmn,

where *μ *is the overall grand mean, *κ_i _*is the fixed effect of the karyotype (*i *=1, 2, 3), *C_j(i) _*is the random effect of the *j*th cross (*j *= 1, 2, ⋯, 6) within karyotype *i*, *τ_k _*is the fixed effect of the developmental temperature (18°C or 22°C), *ι_l _*is the fixed effect of inbreeding (isogenic or outbred homokaryotypic flies), *ς_m _*is the fixed effect of sex, and *ε_ijklmn _*is the residual error associated with the thermal preference (*T*_p_) of the *n*th fly from the *m*th sex with the *i*th karyotype from the *j*th cross that was derived from the *ι*th group of crosses and assayed at the *k*th temperature. The covariate plate-hour was also introduced in the model to control for differences in circadian activity since several trials were conducted during each day. A similar linear model was used for knock out temperature, also introducing water-bath as a covariate since *T*_ko _was assessed in different water-baths.

Notice that for the main effect "karyotype" the linear model (1) can be conveniently reduced to the following two-level nested ANOVA model:

(2) $$T_{\text{p}\left(ijk\right)}=\mu +\kappa_{i}+C_{j(i)}+e_{ijk}\text{,}$$

where the sum of squares for the error term *e_ijk _*is simply the sum of the sum of squares for the remainder terms in (1). The usefulness of this model reduction is to efficiently perform randomization tests to test the null hypothesis about karyotype effects in a randomized (i.e., random assignment) experiment [80]. Permutation tests are far less sensitive to the presence of outliers than parametric tests. The null hypothesis of no karyotype effect was tested here after performing random permutations among replicate and selection temperature for the among selection temperature *F*-statistics. Each test used 10,000 random permutations.

#### b) Karyotype variation

To asses the effect of O chromosome karyotypes on *T*_p _and *T*_ko _we have focused in the outbred crosses, including both structural homo- and heterokaryotypes. The linear model used was similar to (1) including the fixed effect of karyotype (*κ_i_*; *i *= 1, 2, ⋯, 6), the random effect of cross within karyotypes (*C_j(*i*)_*; *j *= 1, 2, ⋯, 6), the fixed effect of developmental temperature, and the fixed effect of sex. The covariate plate-hour was also introduced in the model. As above, a similar linear model was used for knock out temperature, also introducing water-bath as a covariate.

In the original Palaearctic populations chromosome arrangements O_3+4 _and O_3+4+8 _have a higher frequency at lower latitudes than arrangement O_st_, and the converse is true a higher latitudes [35,36]. For this reason, the variation explained by the six karyotypes was further decomposed after pooling the first two arrangements into a single class (${\text{O}}_{3+4}^{*}$) as follows: between the two ${\text{O}}_{\text{st}}/{\text{O}}_{3+4}^{*}$ heterokaryotypes; among the three ${\text{O}}_{3+4}^{*}/{\text{O}}_{3+4}^{*}$ karyotypes; and among O_st_/O_st_, ${\text{O}}_{\text{st}}/{\text{O}}_{3+4}^{*}$, ${\text{O}}_{3+4}^{*}/{\text{O}}_{3+4}^{*}$. The karyotypic values for *T*_p _and *T*_ko _were also estimated in the additive-dominance scale [81,82] after pooling the two chromosome arrangements that share O_3+4 _(each comparison or contrast between two means has one degree of freedom).

The genetic correlation between *T*_p _and *T*_ko _can be approached as indicated in Betrán et al. [26]. Assuming that the components of the between karyotypes sums of squares and cross-products (SSCP) hypothesis matrix (**H***_k_*) are entirely genetic in origin, the correlation coefficient between the means of all six outbred karyotypes is given by:

(3) $$r_{k}=\frac{{\text{H}}_{k}\left(1,2\right)}{\sqrt{{\text{H}}_{k}\left(1,1\right){\text{H}}_{k}\left(2,2\right)}},$$

where **H***_k _*(1, 2) is the off-diagonal element (sum of products of karyotype averages), and **H***_k _*(*i*, *i*) is a diagonal element (sum of squares of karyotypes averages) for the *i*th variable. This correlation coefficient is obviously an approximation to the genetic correlation because the **H***_k _*matrix also contains a fraction of the variation among the isogenic lines used to obtain the outbred flies (see Experimental settings). The correlation coefficient can be tested as:

(4) $$t=r\sqrt{\frac{k-2}{1-r^{2}},}$$

where *k *is the number of karyotypes [83]. After pooling the arrangements that share arrangement O_3+4 _into a single class, we can now obtain the new hypothesis matrix **H***_p_*. The correlation coefficient between the pooled averages can be estimated as:

(5) $$r_{p}=\frac{{\text{H}}_{p}\left(1,2\right)}{\sqrt{{\text{H}}_{k}\left(1,1\right){\text{H}}_{k}\left(2,2\right)}}.$$

The square of this correlation can be interpreted as that fraction of the total variation among karyotypes that is explained by O_st_/O_st_, ${\text{O}}_{\text{st}}/{\text{O}}_{3+4}^{*}$, ${\text{O}}_{3+4}^{*}/{\text{O}}_{3+4}^{*}$.

#### c) Computer software for statistical analysis

The computer programs used for statistical data analyses were MATLAB algebra program environment (ver. 7.0.4 [84]) together with the collection of tools supplied by the Statistics Toolbox. The statistical software packages STATISTICA version 9 [85] and SPSS version 15 [86] were also used.

## Authors' contributions

OD, GC, JB, MP and MS sampled flies from the natural population and setup isofemale lines. OD and GC obtained the isochromosomal lines. JB helped in gene arrangement identification. GC and MP assayed the isochromosomal lines for microsatellites. OD, CR and GC carried out experimental crosses and egg collections. OD, GC, JB, and MS assayed the flies for thermal preference and knock out temperature. OD, CR, ELR and MS conceived the study. OD, CR, and MS carried out statistical analyses and drafted the manuscript. All authors read and approved the final manuscript.
